# Pragmatic randomized controlled trials: strengthening the concept through a robust international collaborative network: PRIME-9—Pragmatic Research and Innovation through Multinational Experimentation

**DOI:** 10.1186/s13063-024-07935-y

**Published:** 2024-01-23

**Authors:** Elmir Omerovic, Mark Petrie, Björn Redfors, Stephen Fremes, Gavin Murphy, Guillaume Marquis-Gravel, Alexandra Lansky, Eric Velazquez, Divaka Perera, Christopher Reid, Julian Smith, Peter van der Meer, Eric Lipsic, Peter Juni, John McMurray, Johann Bauersachs, Lars Køber, Jean L. Rouleau, Torsten Doenst

**Affiliations:** 1 Department of Cardiology, Sahlgrenska University Hospital, Institute of Medicine, Department of Molecular and Clinical Medicine, Sahlgrenska Academy at University of Gothenburg, Bruna Stråket 16, 41345 Gothenburg, Sweden; 2 British Heart Foundation Cardiovascular Research Centre, University of Glasgow, Glasgow, Scotland, UK; 3 Department of Surgery, University of Toronto, Toronto, ON Canada; 4 Sunnybrook Health Sciences Centre, Toronto, ON Canada; 5 Sunnybrook Research Institute, Toronto, ON Canada; 6 Cardiovascular Research Centre, University of Leicester, Glenfield Hospital, Groby Road, Leicester, LE3 9QP UK; 7 Montreal Heart Institute, Université de Montréal, Montreal, QC Canada; 8 Division of Cardiovascular Medicine, Department of Internal Medicine, Yale School of Medicine, New Haven, CT USA; 9 British Heart Foundation Centre of Research Excellence and National Institute for Health and Care Research Biomedical Research Centre at the School of Cardiovascular and Metabolic Medicine and Sciences, King’s College London, London, UK; 10 Curtin School of Population Health, Faculty of Health Sciences, Curtin University, Kent Street, Bentley, WA 6102 Australia; 11 Department of Surgery (School of Clinical Sciences at Monash Health), Monash University, Melbourne, VIC Australia; 12 Department of Cardiothoracic Surgery, Monash Health, Melbourne, VIC Australia; 13 Department of Cardiology, Center for Blistering Diseases, University of Groningen, University Medical Center Groningen, Groningen, the Netherlands; 14 Department of Cardiology, University Medical Center Groningen, University of Groningen, Hanzeplein 1, PO Box 30.001, 9700 RB Groningen, the Netherlands; 15 Oxford Population Health, University of Oxford, Richard Doll Building, Old Road Campus, Oxford, OX3 7LF UK; 16 Department of Cardiology and Angiology, Hannover Medical School, Hannover, Germany; 17 Department of Cardiology, Rigshospitalet, Copenhagen University Hospital, Copenhagen, Denmark; 18 Institut de Cardiologie de Montréal, Université de Montréal, Montréal, Canada; 19 Department of Cardiothoracic Surgery, Friedrich-Schiller-University Jena, University Hospital, Jena, Germany

## Abstract

In an era focused on value-based healthcare, the quality of healthcare and resource allocation should be underpinned by empirical evidence. Pragmatic clinical trials (pRCTs) are essential in this endeavor, providing randomized controlled trial (RCT) insights that encapsulate real-world effects of interventions. The rising popularity of pRCTs can be attributed to their ability to mirror real-world practices, accommodate larger sample sizes, and provide cost advantages over traditional RCTs. By harmonizing efficacy with effectiveness, pRCTs assist decision-makers in prioritizing interventions that have a substantial public health impact and align with the tenets of value-based health care. An international network for pRCT provides several advantages, including larger and diverse patient populations, access to a broader range of healthcare settings, sharing knowledge and expertise, and overcoming ethical and regulatory barriers. The hypothesis and study design of pRCT answers the decision-maker’s questions. pRCT compares clinically relevant alternative interventions, recruits participants from diverse practice settings, and collects data on various health outcomes. They are scarce because the medical products industry typically does not fund pRCT. Prioritizing these studies by expanding the infrastructure to conduct clinical research within the healthcare delivery system and increasing public and private funding for these studies will be necessary to facilitate pRCTs. These changes require more clinical and health policy decision-makers in clinical research priority setting, infrastructure development, and funding. This paper presents a comprehensive overview of pRCTs, emphasizing their importance in evidence-based medicine and the advantages of an international collaborative network for their execution. It details the development of PRIME-9, an international initiative across nine countries to advance pRCTs, and explores various statistical approaches for these trials. The paper underscores the need to overcome current challenges, such as funding limitations and infrastructural constraints, to leverage the full potential of pRCTs in optimizing healthcare quality and resource utilization.

## Introduction

Randomized controlled trials (RCTs) are highly prioritized in health care and clinical research as they are a valuable source of information within evidence-based medicine (EBM). One type of RCT that has gained attention in recent years is the pragmatic randomized controlled trial (pRCT), which aims to evaluate interventions in real-world settings with diverse patient populations and a wide range of healthcare providers [1, 2]. The focus of pRCT is on assessing interventions’ effectiveness rather than their efficacy in controlled experimental conditions. This can help bridge the gap between research findings and their application in clinical practice. Establishing robust international collaborative networks can strengthen the concept of pRCT and promote their use in clinical research. Such a network comprises researchers, clinicians, patients, and policymakers from different countries, who work together to design and conduct pRCT that addresses important clinical questions and provides practical solutions for patients and healthcare providers. Notably, the financing of these pRCT also can come from a diverse range of sources outside of the usual routes of industry funding or charity or national health services funding. Collaboration with philanthropic organizations or patient advocacy groups in pRCTs can help to beautifully align research with patient needs and concerns, providing a delicate balance to the commercial interests of industry or research agendas of government agencies. This thoughtful approach can foster patient-centered research, enhancing the relevance and applicability of results to a wider population.

The role of industrial funding in RCTs has raised concerns about potential conflicts of interest and bias in study design and reporting [3, 4]. Approximately 70–80% of RCTs are financed by industry sources, which may prioritize positive results that benefit the company’s interests [5–7]. Ensuring a diverse range of funding sources for RCTs is essential to promote transparency, objectivity, and the best interests of patients and societies. By promoting a diverse range of funding sources for pRCT, we can also increase the number and variety of studies conducted. This can lead to a better understanding of a broader range of interventions in distinct patient populations, improving the applicability and relevance of research findings. Establishing a robust international collaborative network for pRCT, funded by various sources outside the medical-industrial complex, can help ensure that clinical research is more patient-centered, transparent, and objective. Some advantages and disadvantages of industrial funding in RCTs are listed in Table 1. This paper presents a comprehensive overview of pRCTs, emphasizing their importance in evidence-based medicine and the advantages of an international collaborative network for their execution. It details the development of PRIME-9, an international initiative across nine countries aimed at advancing pRCTs, and explores various statistical approaches for these trials. The paper underscores the need to overcome current challenges, such as funding limitations and infrastructural constraints, to leverage the full potential of pRCTs in optimizing healthcare quality and resource utilization.

**Table 1:** Advantages and disadvantages of industrial funding in RCTs

Definition	Advantages	Disadvantages
Non-industry-funded trials are clinical studies or trials funded by sources other than the pharmaceutical, medical device, or biotechnology industries. These sources may include academic institutions, government agencies, non-profit organizations, or individual donors	Answer questions that the industry may not be willing to or is uninterested to study Answer questions of interest to clinicians and patients Less influence of industry on study design and conduct Less concern for commercial interests Potentially lower risk of publication bias Team building across countries	Reliability of endpoints other than all-cause mortality Limited financial resources Limited access to specialized equipment or technologies Smaller sample size due to limited funding Fewer resources for marketing and disseminating results Often does not follow the standard required by FDA/EMA Difficulty in recruiting participants due to limited resources Legal and ethical issues regarding data transfer across borders

## What are pragmatic clinical trials, and why are they essential for evidence-based medicine?

**Fig. 1:**
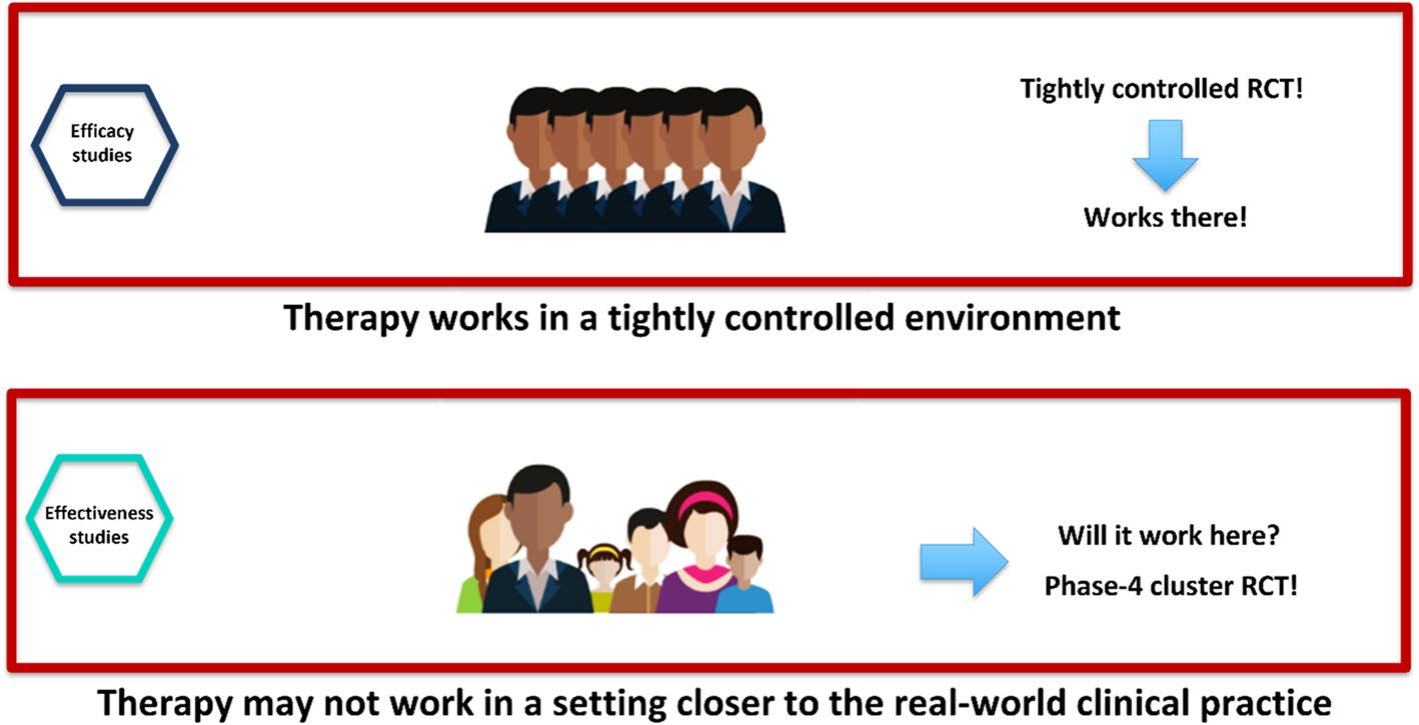
Efficacy versus effectiveness. The illustration depicts the contrast between a classical randomized controlled trial (RCT), which is rigorously controlled and focused on internal validity (efficacy), and a pragmatic RCT, which is intended to mirror clinical practice in the real world and optimized for external validity (effectiveness). When assessing the effectiveness and safety of interventions, healthcare decision-makers must consider both types of trials. Classical RCTs furnish valuable insights into an intervention’s efficacy in a highly controlled environment, while pragmatic RCTs yield insights into an intervention’s real-world performance, potentially offering more comprehensive generalizability. If an intervention proves ineffective in terms of its effectiveness, decision-makers may need to consider prioritizing healthcare resources based on the ethical principle of justice

pRCTs are RCTs that aim to test the effectiveness of healthcare interventions in real-world settings. pRCTs are designed to evaluate the effectiveness of an intervention in a diverse patient population, using broad eligibility criteria and minimal exclusion criteria. The goal of a pRCT is to determine whether an intervention is effective in routine clinical practice, as opposed to a highly controlled and specialized clinical trial setting (Table 2 and Fig. 1). pRCTs are becoming increasingly popular because they offer several advantages over traditional RCTs (Table 2). First, pRCTs are more representative of real-world practice, which can lead to better generalizability of the results. Second, pRCT typically has a larger sample size than traditional RCTs, which can improve the power and precision of the results. Third, pRCTs are often more cost-effective than conventional RCTs since they are designed to be conducted within the routine healthcare system. One key consideration in pRCT is the balance between efficacy and effectiveness. Efficacy refers to the ability of an intervention to produce a desired effect under ideal conditions.

**Table 2:** Advantages and disadvantages of pragmatic RCTs over traditional RCTs

Definition	Advantages	Disadvantages
A pragmatic clinical trial is a study designed to evaluate an intervention’s effectiveness in real-world conditions, reflecting the specific patient population and usual clinical practice. A pragmatic clinical trial aims to provide evidence that can be directly applied to clinical decision-making	Reflects real-world conditions Results are directly applicable to clinical decision-making Involves a diverse patient population May be more cost-effective May provide information on unexpected benefits or harms of interventions May reduce the potential for selection bias More generalizable to broader populations	May require larger sample sizes May require more extended follow-up periods May have limited ability to evaluate complex interventions May have limitations in outcome measurement and data collection

In contrast, effectiveness refers to the ability of an intervention to produce a desired effect in real-world settings (Fig. 1). Because pRCT aims to evaluate interventions in routine clinical practice rather than highly controlled and specialized clinical trial settings, the emphasis is on both effectiveness and adverse events. By focusing on effectiveness and adverse events, pRCT can provide decision-makers with valuable information about the real-world impact of healthcare interventions. Another vital consideration in pRCT is the prioritization of interventions. In a resource-constrained healthcare system, decision-makers must prioritize interventions based on their potential impact on public health. pRCT can help decision-makers identify interventions most likely to significantly impact public health by testing them in diverse patient populations and real-world settings. pRCT can test established interventions and challenge the status quo in health care. By prioritizing interventions that are effective in pRCT, decision-makers can allocate resources more effectively and improve patient’s overall quality of care. Some examples of successful pRCTs that influenced clinical practice and that were easily generalizable to real-world clinical care are registry randomized controlled trials performed in Sweden within the SWEDEHEART registry platform, including TASTE, VALIDATE, iFR, and DETOX [8–11].

pRCTs can serve as a crucial bridge by providing immediately relevant and applicable evidence to clinical settings to address the implementation concerns and delays in integrating benefits into routine clinical practice. Pragmatic RCTs are uniquely positioned to ease the transition from trial to treatment due to their design attributes. They encompass broad eligibility criteria, reflecting the diverse patient populations in everyday practice. Doing so yields findings that can be generalized to a broader patient base than classical RCTs. This generalizability can enhance the confidence of healthcare decision-makers and providers in adopting the interventions since the results are more representative of actual clinical scenarios. Moreover, pRCTs can facilitate more rapid integration of research findings into clinical practice by involving a wide range of healthcare stakeholders, including clinicians and patients, during the research process. Their input can help ensure that the interventions tested are theoretically effective and practically feasible, addressing common barriers to implementation such as workforce training, resource allocation, and patient compliance.

## International collaboration

Collaborations between several nations are becoming more widespread in clinical research to overcome pRCT issues. Collaborations across nations can expand trial sample size, minimize variability, and improve the generalizability of findings.

This paper elucidates a unified, cross-country approach to executing pragmatic clinical trials, mainly focused on major adverse cardiovascular events. It delineates how these trials are seamlessly conducted across diverse geographic locales, harnessing the strength of international collaboration in cardiovascular research. We focus on Sweden, Canada, Denmark, the UK, the USA, Netherlands, Germany, Australia, and New Zealand, which have extensive experience conducting clinical trials and most of which have established registry platforms that can facilitate collaboration.

## Advantages of an international collaborative network for pRCT

An international network for pragmatic clinical trials can provide several advantages over conducting pRCT within a single country. Firstly, an international network for pragmatic clinical trials can enable the recruitment of more extensive and diverse patient populations. By recruiting patients from multiple countries, pRCT can include a broader range of patient populations, increasing the generalizability of study results. This can be particularly important for interventions affecting patients from different cultures or healthcare systems. Secondly, an international network for pragmatic clinical trials can provide access to a broader range of healthcare settings. By including healthcare settings from multiple countries, pRCT can evaluate the effectiveness of interventions in various real-world settings, which can improve the external validity of study results. This can be particularly important for interventions with different effects in different healthcare settings, such as primary versus hospital-based care. Thirdly, an international network for pragmatic clinical trials can facilitate the sharing of knowledge and expertise across countries. By collaborating with researchers, healthcare professionals, and clinical trial units from multiple countries, pRCT can leverage the expertise and resources of each country to improve study design, implementation, and interpretation. This can lead to more efficient and effective pRCT and the development of best practices for conducting pRCT in different healthcare systems. Finally, an international network for pragmatic clinical trials can help to overcome ethical and regulatory barriers to conducting pRCT. By working together across multiple countries, pRCT can navigate the complex ethical and regulatory issues that can arise when conducting research in different healthcare systems. This can lead to more streamlined and efficient study processes and greater consistency in study protocols and outcomes.

## Challenges in pRCTs

While pRCTs offer several positive aspects, they also present some challenges. One challenging aspect of pRCTs is that they often require high collaboration between healthcare providers and researchers. Conducting pRCTs in real-world settings requires the participation of multiple stakeholders, including healthcare providers, patients, and healthcare systems. This can make pRCTs more complex and challenging to implement than traditional RCTs, often conducted in specialized clinical trial settings. Another challenge is that they can be more difficult to develop and conduct than typical RCTs. Because pRCTs are conducted in real-world settings, they frequently necessitate rigorous planning and execution to guarantee that the intervention is delivered uniformly across diverse healthcare settings.

Additionally, pRCTs may confront ethical complexities, necessitating a balance between the study’s scientific rigor and the feasibility of conducting interventions in real-world contexts. The need to accommodate more diverse patient groups, such as those with comorbidities, might require easing eligibility criteria. Nevertheless, this broadens the scope of applicability as results become more relevant to populations typically omitted from conventional RCTs. Data quality and reliability present further challenges for pRCTs as they often depend on data from everyday clinical practice and dedicated national quality-of-care registries. Establishing minimum data quality standards for the collaborative can help mitigate these anticipated data quality issues.

In the field of pRCT, a robust tool known as PRECIS-2 has been recently introduced [12]. The PRECIS-2 tool, standing for “"Pragmatic Explanatory Continuum Indicator Summary 2,” aids trialists in designing and implementing their trials in line with the intended study objectives. In the context of the PRIME-9 initiative, the PRECIS-2 tool provides robust guidance for trialists to align their trial designs with their study’s purpose. It has been validated and improved by over 80 international experts and covers multiple domains focusing on the practical applicability of trial results. Factors like eligibility, recruitment, setting, organization, flexibility, follow-up, primary outcome, and analysis shape this applicability. A higher PRECIS-2 score suggests a pragmatic approach. Trialists are guided to consider factors such as geographic location, healthcare system, socioeconomic status, and ethnicity when assessing the applicability of trial results. They are also reminded to consider available resources in usual care and to ensure the primary outcome relevance to patients and care commissioners. The tool encourages consistency in decision-making and promotes the development of evidence-based care. Moreover, it emphasizes the need for external validity assessment before starting a study, affirming the importance of pragmatism in trial design and conduct for achieving this validity.

While the previous sections of this manuscript have highlighted the numerous advantages of an international collaborative network for conducting pragmatic pRCTs, including the potential for enhanced generalizability and the ability to capture a broader range of population data, it is equally important to acknowledge the intrinsic challenges posed by such endeavors. Different healthcare system structures can lead to variability in the effectiveness of interventions between countries. This is often a reflection of the disparate healthcare delivery models, funding mechanisms, and patient populations, which can influence both the trial outcomes and the feasibility of implementation post-trial. Furthermore, the logistics of setting up multicenter trials across diverse regulatory environments can introduce significant delays. This affects the data collection timeline and can lead to staggered implementation of interventions due to the asynchrony in trial initiation between different sites. “Treatment as Usual” (TAU) as the control condition is particularly challenging in multicountry pRCTs, given TAU can vary substantially between different settings. These variations must be carefully considered when designing the study and interpreting the results, as they can impact the trial's internal and external validity. Additionally, the network of locations represented in our study is limited to developed and Westernized countries, which does not capture the healthcare realities of low- to middle-income countries or non-Westernized settings. This limitation has implications for the generalizability of the results and highlights the need for a more inclusive approach to constructing international collaborative networks for pRCTs. Despite these challenges, we believe that the strategies proposed in this manuscript, such as standardization of protocols, adaptive trial designs, and stakeholder engagement, can help to mitigate these issues. For instance, adopting core outcome sets and harmonizing data collection standards can address differences in healthcare systems and TAU across countries. Moreover, proactive engagement with regulatory bodies can help expedite trial setup and align implementation timelines more closely between different locations.

Conducting trials in multiple countries can present challenges such as language and cultural barriers, differences in regulatory and ethical frameworks, and healthcare systems. For example, Sweden and the USA provide distinct cases of how national particularities can impact clinical research. Clinical research is shaped by national variations, as seen in Sweden’s centralized medical review and the US diverse Institutional Review Board (IRB) system. Sweden’s uniform review expedites multicenter trials, while the USA faces variable IRB outcomes, affecting trial starts. Privacy laws also vary, with Sweden applying stringent General Data Protection Regulation rules and the USA following Health Insurance Portability and Accountability Act regulations. Healthcare systems further complicate the conduction of trials; Sweden’s universal coverage facilitates recruitment and application of findings, contrasting the US’s more complex, insurance-based model, which impacts participant accessibility and the diversity of clinical trials. Sweden has taxpayer-funded healthcare that ensures all residents can access healthcare services with minimal out-of-pocket costs. This universal access can influence clinical trial designs by potentially making it easier to recruit participants and apply findings broadly across the population, as most barriers related to healthcare coverage are minimized. In contrast, the healthcare system in the USA is a mix of public and private insurance, with a significant portion of healthcare costs covered by private insurance companies. This fragmented system can create challenges in clinical trials concerning participant recruitment, diversity, and applicability of findings, as there may be significant differences in access to healthcare services and treatments.

## Statistical approaches for pRCTs

### Frequentist statistics

Frequentist statistics have been the dominant approach in analyzing RCT data for many years. This approach involves making inferences about a population based on the properties of a sample, assuming that the sample was randomly drawn from the population. Frequentist methods provide estimates of parameters, such as means or proportions, and their associated standard errors, which can be used to make inferences about differences between groups or the effects of interventions. Statistical significance testing in the design of RCTs with frequentist statistics is focused on reducing the likelihood of making a type I mistake (rejecting a valid null hypothesis). This is accomplished by establishing a predetermined significance threshold, often 0.05, and comparing the *p*-value, or the likelihood of receiving a result as extreme or more extreme than the actual result, to this level. If the *p*-value is less than the significance level, the null hypothesis is rejected, and the result is considered statistically significant. However, frequentist statistics have been criticized for their limited ability to provide information about the probability of a hypothesis being true or to quantify the uncertainty around estimates of parameters. The dichotomous answer to a research question provided by a frequentist approach often does not take into consideration the nuances of the treatment effects. Bayesian statistics, an alternative approach to statistical inference, can provide this information but are less commonly used in RCTs. Overall, frequentist statistics remain the dominant approach in RCTs due to their ease of use, established analysis methods, ability to control type-I error rates, and familiarity of researchers and clinicians with the approach. However, there is increasing interest in exploring alternative approaches to statistical inference, such as Bayesian methods, that may provide additional insights into the data generated by RCTs.

### Bayesian statistics

**Fig. 2:**
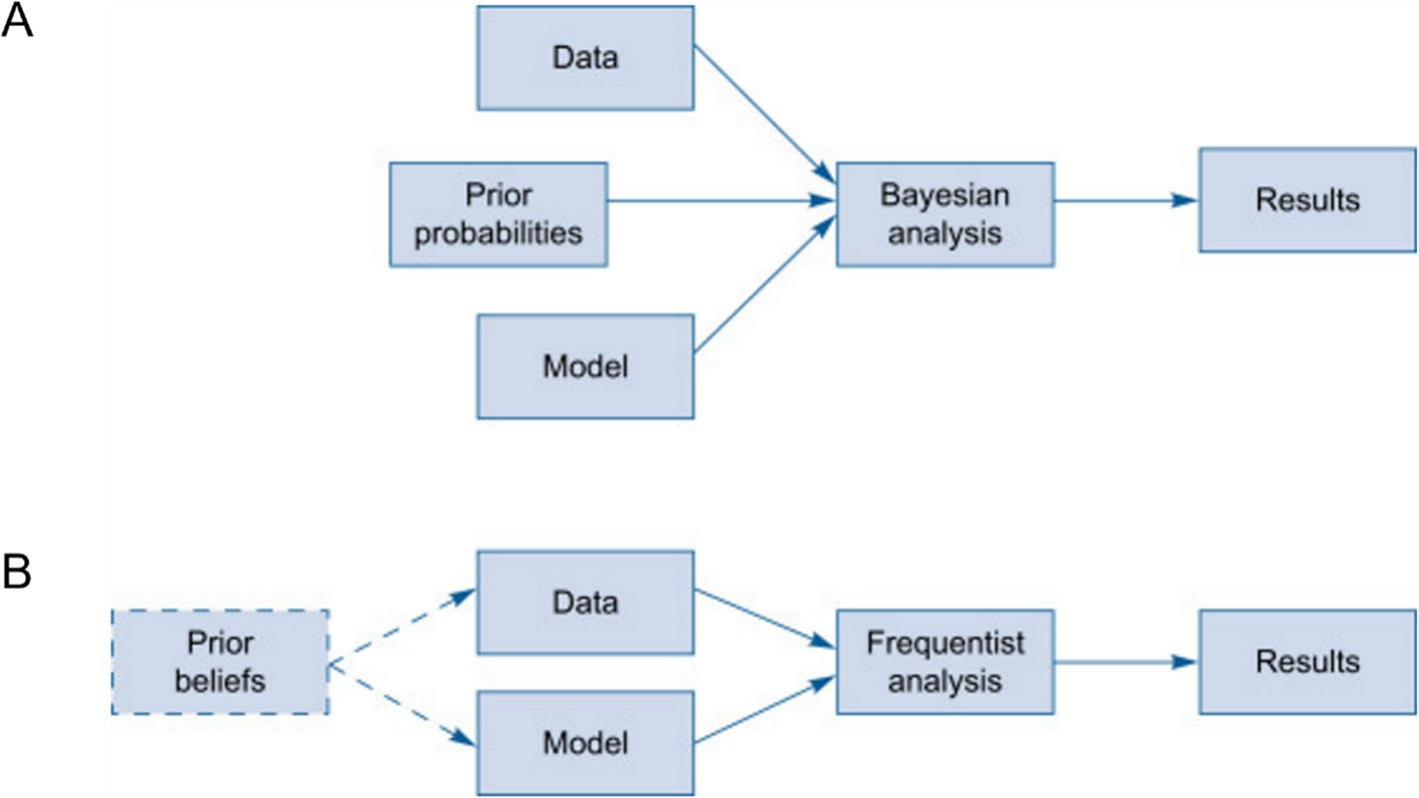
Comparison of Bayesian and frequentist statistics. **A** Bayesian analysis workflow—this panel illustrates the essential components of Bayesian analysis, including the incorporation of prior probabilities, the calculation of likelihood based on observed data, and the computation of posterior probabilities using Bayes’ theorem. The iterative nature of Bayesian updating is also depicted, highlighting the adaptability of the approach as new evidence becomes available. **B** Frequentist analysis workflow—in contrast, this panel demonstrates the frequentist analysis workflow, which involves estimating parameters based solely on the likelihood of observed data. The focus is on hypothesis testing and the calculation of *p*-values, without the incorporation of prior knowledge

Bayesian statistics has gained prominence in medical research and practice. Central to this framework are the concepts of prior beliefs (prior probability), likelihood, posterior probabilities, and Bayesian updating, which allow for incorporating prior knowledge and updating beliefs based on new evidence (Fig. 2). Here are the definitions for the central components of the Bayesian statistical framework. *Prior probability:* The foundation of Bayesian analysis lies in utilizing prior probabilities, representing initial beliefs about the parameters of interest before any new data is observed. Priors in Bayesian analysis, which can be informed by previous research, expert judgment, or plausible assumptions, play a crucial role in guiding the analysis. These prior probabilities vary in the degree of information they convey: they can be informative, weakly informative, or non-informative. While informative priors integrate substantial prior knowledge and can be powerful in guiding the analysis, their adoption in randomized controlled trials (RCTs) is cautious and not broadly established. This is due to concerns regarding introducing bias and a preference for priors that minimize prior influence in RCTs. Non-informative or weakly informative priors are more commonly favored, as they are designed to exert minimal influence on the analysis, thus supporting a more conservative and objective assessment of the data collected in the trial. *Likelihood:* Likelihood represents the probability of observing the data given a particular set of parameter values. In medical research, this might involve determining the likelihood of observing a specific treatment effect given a particular treatment regime. *Posterior probability:* The combination of prior probabilities and likelihood gives rise to the posterior probabilities, representing the updated beliefs about the parameters of interest after considering the new evidence. This is achieved by applying Bayes’ theorem, which effectively weighs the prior probabilities against the likelihood of the observed data. Different sets of posterior probabilities can be generated incorporating different strengths of prior probabilities, which facilitates the interpretation of the findings for clinicians with different a priori opinions about the treatment effect. *Bayesian updating:* A key advantage of the Bayesian approach is its ability to update beliefs as new evidence becomes available iteratively, without statistical penalty. This dynamic nature enables the continual refinement of knowledge and the efficient incorporation of accumulating evidence from multiple sources.

Bayesian statistical frameworks allow for more complex and nuanced models which can adapt to an evolving data landscape. This adaptability is particularly relevant when there are issues like missing data, changes in trial endpoints, or emergent subgroups of interest, which require a dynamic analytical approach. Moreover, the probabilistic nature of Bayesian inference provides more intuitive results that can be directly used for decision-making purposes. For instance, clinicians, policy-makers, and patients would find it accessible to understand the probability of a new drug being at least 5% better than the existing standard treatment. Such straightforward interpretations can greatly facilitate the translation of clinical trial data into practical medical decisions.

Bayesian statistics has significant advantages over the classic frequentist technique for conducting pRCT to evaluate therapies in real-world settings with heterogeneous patient populations [13, 14]. One of the critical advantages of Bayesian statistics is the ability to incorporate prior knowledge (RCT, observational studies, and/or biological plausibility based on mechanistic studies) and beliefs (expert opinions) about the intervention being studied [15, 16]. Prior knowledge can help inform the trial design and analysis [17, 18]. By incorporating prior knowledge and beliefs, researchers can make more informed decisions about the trial’s design and increase the trial’s efficiency by reducing the sample size needed to detect a significant effect. Bayesian methods allow for flexible sample size determination, which can be helpful in pragmatic trials where it may be difficult to recruit large numbers of participants [13, 19]. Bayesian sequential designs have become increasingly popular in clinical trials for their ability to optimize resource allocation and achieve greater statistical power compared to standard fixed sample size designs [14, 15, 19–21]. One of the key benefits of Bayesian approaches is their ability to provide direct measures of evidence on a clinical scale, leading to more meaningful interpretations of trial outcomes. In contrast, traditional frequentist methods rely on *p*-values and hypothesis testing, which may not be as relevant in a clinical context. An additional advantage of Bayesian methods is their capacity to generate reliable results, distinct from the framework of type I error [22]. This is due to their focus on the posterior probability of the treatment effect given the data rather than the probability of observing the data given a specific hypothesis. Bayesian methods also offer the flexibility to conduct interim analyses and frequent monitoring of accumulating data without requiring advance planning or statistical penalty for multiple testing [23]. By allowing for early stopping or modifications of the trial design based on emerging results, this approach can save time and resources while also increasing the power of the trial. Furthermore, Bayesian methods can stop studies for futility, harm, or efficacy earlier, providing a more ethical approach to clinical trials. This is because Bayesian analyses provide simultaneous probability statements regarding multiple outcomes without statistical penalty for multiple comparisons enabling a more comprehensive data analysis.

In modern cardiology, the ORBITA-2 [24] and ISCHEMIA [25] trials stand out as significant randomized controlled trials (RCTs) incorporating Bayesian methodology. ORBITA-2 is a pragmatic trial that utilizes Bayesian techniques to evaluate the clinical impact of adding percutaneous coronary intervention to optimal medical therapy in patients with stable coronary artery disease and moderate-to-severe ischemia. Reflecting a pragmatic approach, ORBITA-2 features inclusive eligibility criteria and focuses on outcomes that resonate with clinical realities. The Bayesian framework adopted by the trial enhances flexibility in interim analysis and enables an adaptive design, thereby optimizing the trial process. Contrastingly, the ISCHEMIA trial represents a more traditional, non-pragmatic trial structure but also benefits from Bayesian methods. The ISCHEMIA trial—a study comparing the outcomes of medical therapy with and without invasive interventions in patients with stable ischemic heart disease—applies Bayesian statistics to facilitate refined interpretations of both primary and secondary endpoints. Bayesian methods in this context provide the advantage of assimilating prior knowledge and adaptively refining probabilistic assessments with the emergence of new data throughout the trial.

## Platform pragmatic clinical trials

**Fig. 3:**
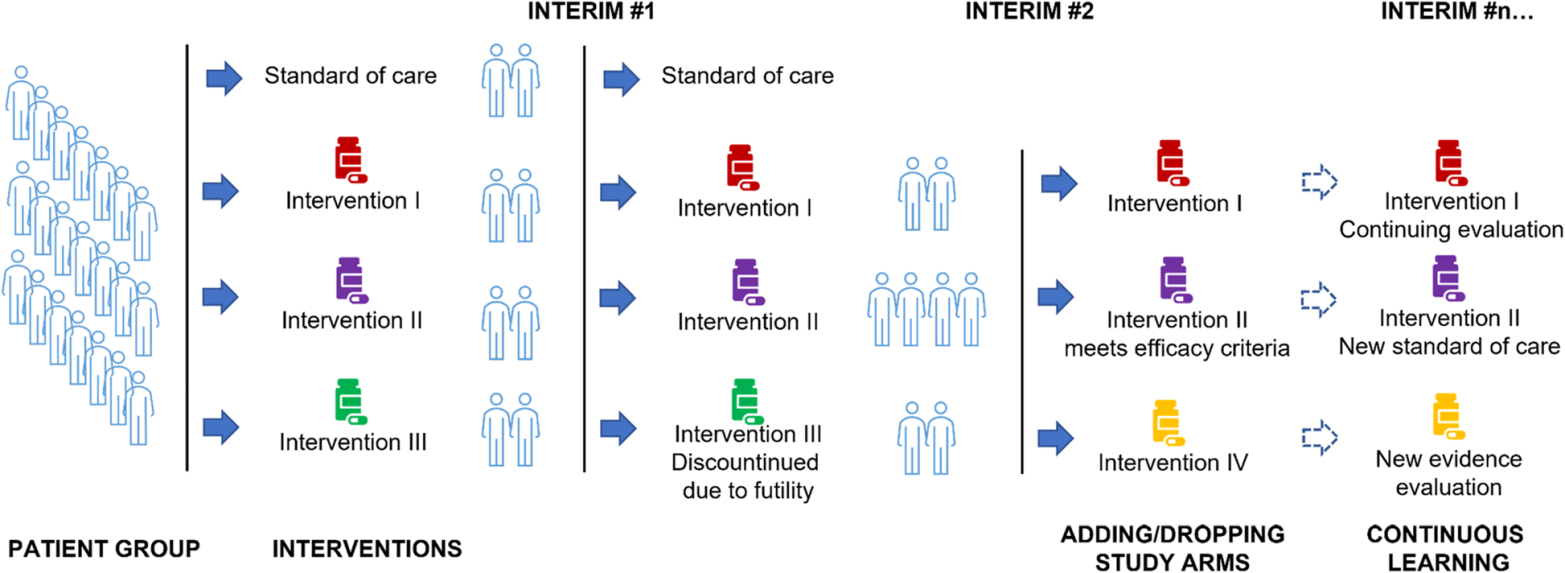
Organization and advantages of the platform pragmatic clinical trials. The potential next step in developing the PRIME-9 collaboration will be the organization and advantages of platform pragmatic clinical trials (ppRCT). Platform trials can test multiple interventions simultaneously within a single trial, allowing for the streamlined development of effective therapies and the ability to adapt to changing scientific knowledge. These trials would follow the principles of pragmatic trials, focusing on patient-centered outcomes, efficiency, and flexibility. By conducting ppRCT, the PRIME-9 collaboration can evaluate the effectiveness of multiple interventions for a particular health condition or disease cost-effectively and adaptively. The addition of ppRCT would represent a significant advancement for the PRIME-9 collaboration in their mission to improve patient outcomes and advance healthcare practice worldwide

The next step in developing the PRIME-9 collaboration will be to design and conduct a platform trial [20, 26, 27]. Platform trials are a type of clinical trial designed to test multiple treatments simultaneously in a single study, allowing for more efficient use of resources and reducing the overall time and cost required to bring new therapies into clinical practice (Fig. 3) [21]. Platform trials offer several advantages over traditional clinical trial designs, including:

- Increased efficiency: Platform trials allow multiple treatments to be evaluated simultaneously, reducing the time and cost required to establish new therapeutic standards and guidelines. This can lead to more rapid and cost-effective drug development.
- Flexibility: Platform trials are designed to be flexible and adaptable, allowing new treatments to be added or removed from the trial as they become available. This can help ensure the trial remains relevant and up-to-date as new therapies are developed.
- Reduced sample size: Platform trials can reduce the sample size required to evaluate multiple treatments, as patients can be shared between treatment arms. This can help to reduce the burden on patients and investigators and make the trial more feasible.
- Better control group: Platform trials use a common control group for all treatments, which can help reduce variability in patient populations and increase the validity and generalizability of the study results.
- Improved patient selection: With platform trials employing a master protocol featuring standardized eligibility criteria, there is enhanced precision in patient selection, drawing on relevant and consistent criteria across all treatment arms. This approach not only refines the accuracy of results but also heightens their generalizability. Furthermore, the structure of platform trials potentially allows cost-sharing across treatments under investigation, making the process economically efficient. These factors together enhance the value and impact of the resultant findings.

The REMAP-CAP trial [20] (Randomized, Embedded, Multifactorial Adaptive Platform trial for Community-Acquired Pneumonia), founded on a Bayesian approach, is a prime illustration of the effectiveness of platform RCTs. The REMAP-CAP trial seamlessly expanded its scope in response to the COVID-19 pandemic. Its adaptive nature underlines the efficiency and flexibility inherent in platform trials, particularly exemplified by its capability to rapidly integrate and assess novel interventions for COVID-19. Furthermore, the trial follows pragmatic principles, with broad inclusion criteria inclusive of a diverse patient population and the selection of outcomes that matter most in day-to-day patient care. These aspects expedite the evaluation of therapeutic options and ensure the results are applicable and relevant to a wide clinical practice. The Bayesian methodology facilitates the trial’s adaptability and responsiveness to evolving data, allowing for a dynamic assessment of therapies that aligns closely with real-world healthcare needs. Consequently, REMAP-CAP's design and decision-making processes significantly accelerate the time-to-evaluation of medical interventions, enhancing the relevance and applicability of its results across diverse clinical settings.

## PRIME-9—Pragmatic Research and Innovation through Multinational Experimentation in 9 countries: Sweden, Canada, Denmark, the UK, the USA, Netherlands, Germany, Australia, and New Zealand

**Fig. 4:**
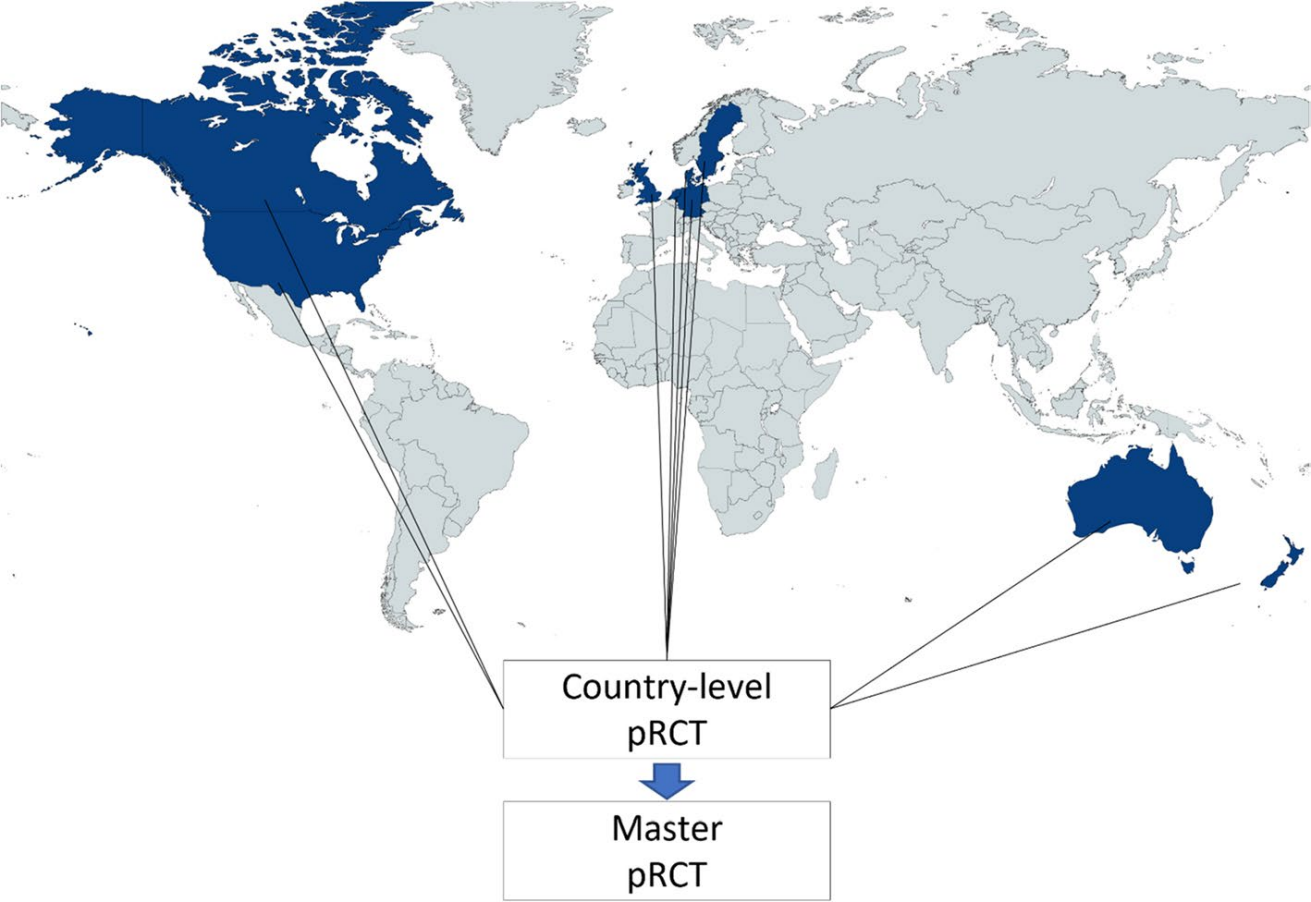
The PRIME-9 collaboration. The PRIME-9 collaboration is an international network of healthcare professionals and researchers who are collaborating to conduct pragmatic clinical trials. This collaboration includes nine countries, namely Sweden, Canada, Denmark, the UK, the USA, Netherlands, Germany, Australia, and New Zealand. The objective of the PRIME-9 collaboration is to investigate vital clinical questions by conducting extensive, real-world studies that represent the diversity of patients and healthcare settings. Pragmatic clinical trials concentrate on the effectiveness of interventions in real-world settings rather than the efficacy of interventions in controlled environments. By conducting these trials, the PRIME-9 collaboration seeks to provide valuable insights into the best practices for treating and managing various health conditions. The partnership includes a multidisciplinary team of researchers, clinicians, and patient advocates working together to design and execute relevant and meaningful studies for patients and healthcare providers. Country-level pragmatic RCTs will be pooled into a master-level RCT, allowing the PRIME-9 collaboration to conduct studies on a larger scale and more efficiently than individual research teams

Collaboration between Sweden, Canada, Denmark, the UK, the USA, Netherlands, Germany, Australia, and New Zealand can become the world’s leading collaborative platform for conducting pRCTs (Fig. 4). Here are the crucial steps outlining how the collaboration was established:

- PRIME-9 is founded on friendship, shared values, a unified vision, trust, flexibility, compromise, effective communication, transparency, ethics, and long-term dedication.
- A solid governance structure and leadership team, with equal representation from each country, is established to ensure a fair decision-making process.
- The first project, STICH 3.0 trial (NCT05761067), comparing coronary artery bypass grafting (CABG) versus percutaneous coronary intervention (PCI) in patients with heart failure with reduced ejection fraction (HFrEF), serves as a visionary but achievable goal and foundation for collaboration.
- A common data platform is developed to adhere to privacy regulations and facilitate sharing.
- Resources such as research infrastructure, study design, and data management tools are shared to reduce costs and increase efficiency.
- Patient representatives are involved in study design and implementation to incorporate the patient perspective and identify patient-centered outcomes.
- Clear communication channels are established, such as regular meetings and online forums.
- Funding is sought from various sources to support research infrastructure, staff salaries, and study implementation, ensuring sustainability.

Due to restricted resources and patient populations in each participating country, conducting large-scale pRCT with all-cause mortality as the primary endpoint in a reasonable time frame is not feasible. The PRIME-9 strategy to pRCT recognizes these limitations. It has adopted a combined endpoint of major adverse cardiovascular events (MACE) across all participating countries to increase the number of events and improve the study’s statistical power. Once the data from the individual studies are collected, the PRIME-9 strategy utilizes Bayesian statistics to pool the data for all-cause mortality—the most important clinical outcome in HFrEF. Bayesian statistics provides a powerful and flexible framework for combining data from different sources, allowing us to incorporate prior information and adjust for heterogeneity between studies. By combining the data in this way, the PRIME-9 strategy can produce a more accurate and precise estimate of the treatment effect, with a narrower confidence interval and a higher level of statistical power.

Furthermore, Bayesian statistics allow for quantifying uncertainty in the data, which is a key aspect of clinical research. The PRIME-9 strategy can use Bayesian methods to calculate the posterior probability of various treatment effects and compare the efficacy of different treatments across the participating countries. It also allows the incorporation of newly generated evidence from the countries when they accrue to modify the posterior probability of the intervention in “real-time.” This allows for a more informed decision-making process in the design and execution of future clinical trials. Costs and speed of patient recruitment are essential considerations in conducting clinical trials, mainly when trials are conducted across multiple countries. PRIME-9 collaboration can help address these challenges by pooling resources and expertise, reducing overall trial costs, and increasing patient recruitment speed. Using registry platforms established in each country can reduce the costs of setting up new trial infrastructure.

Furthermore, by collaborating with national funding resources, we can leverage the available resources in each country to conduct the trials more efficiently. In terms of patient recruitment, collaborations between countries can increase the speed of recruitment by providing access to a larger pool of patients. This can be particularly useful in studies of rare diseases or conditions where patient recruitment can be challenging. Furthermore, the collaborative network between countries can help to ensure that the patient population is more representative, as it is not limited to a single country or region. The PRIME-9 collaboration has the potential to become a leading platform for pRCT. This network aims to generate significant evidence to inform clinical practice and healthcare policy worldwide, ultimately leading to better patient health outcomes. The approach can serve as a model for future collaborations and highlights the importance of sharing resources and expertise to advance clinical research.

In conclusion, pRCT, mainly through international collaboration like the PRIME-9 initiative, holds significant promise in delivering real-world, patient-centered insights that can shape evidence-based medicine. By overcoming current challenges and harnessing diverse resources and expertise, pRCTs can revolutionize our approach to healthcare, optimizing quality and resource utilization for improved patient outcomes worldwide.

## Data Availability

Not applicable.
